# Complete Genome of *Bacillus pumilus* Siphophage Riggi

**DOI:** 10.1128/genomeA.00861-13

**Published:** 2013-12-05

**Authors:** Emily L. Still, Catarina F. Riggi, Karthik R. Chamakura, Gabriel F. Kuty Everett

**Affiliations:** Center for Phage Technology, Texas A&M University, College Station, Texas, USAa; Department of Integrated Science and Technology, James Madison University, Harrisonburg, Virginia, USAb

## Abstract

*Bacillus pumilus* is primarily used in the agricultural industry to promote plant growth and provide resistance to bacterial and fungal plant diseases. It has recently, however, been shown to cause disease in humans. Here, we announce the complete genome of *B. pumilus* phage Riggi.

## GENOME ANNOUNCEMENT

*Bacillus pumilus* is a Gram-positive, spore-forming, soil-inhabiting bacterium. It is used in agriculture as a nitrogen fixer and to promote crop growth (1, 2). It also confers plant resistance to several bacterial and fungal diseases (3). However, cases of food-borne illness and anthrax-like cutaneous lesions have been attributed to the bacteria (4, 5). As *B. pumilus* is commonly found in soil, its pathology could be hazardous to agricultural workers. Here we present the complete genome of *B. pumilus* phage Riggi. The study of *B. pumilus* phages will provide insight into the genetics and physiology of this helpful, yet potentially harmful, bacterium.

*B. pumilus* strain BL-8 was isolated on the campus of James Madison University (6). Riggi was isolated from a soil sample collected in Harrisonburg, VA. Phage DNA was sequenced using 454 pyrosequencing at the Emory GRA Genome Center (Emory University, Atlanta, GA). Trimmed FLX Titanium reads were assembled to a single contig at 36.7-fold coverage using the Newbler assembler, version 2.5.3 (454 Life Sciences), at default settings. Contigs were confirmed to be complete by PCR. Genes were predicted using GeneMarkS (7) and corrected using software tools available on the Center for Phage Technology (CPT) Portal (https://cpt.tamu.edu/cpt-software/portal/).

Riggi has a unit genome of 49,007 bp with a coding density of 92.6% and a GC content of 41.4%. Seventy-eight unique coding sequences were predicted, of which 28 have a predicted function. Analysis of the identified TerL revealed homology to TerLs of phages with long terminal repeats. The terminal repeat of Riggi was determined to be 829 bp by examining the raw sequencing data using the PAUSE (https://cpt.tamu.edu/cpt-software/releases/pause/) method.

Genes encoding proteins related to phage morphogenesis were identified, including those encoding portal protein, minor head protein, scaffold, major capsid protein, tail morphogenesis protein, tape measure protein, the tail completion/head joining protein, and tailspike. A pectin lyase domain was predicted in the putative tailspike. It is hypothesized that the pectin lyase domain is involved in biofilm depolymerization (8). Genes for DNA replication and recombination proteins found were those encoding DNA helicase, primase, polymerase, Holliday junction resolvase, and three nucleases. Riggi also carries genes encoding proteins for DNA biosynthesis (thymidylate synthase and deoxynucleoside monophosphate kinase). The holin and endolysin for phage lysis of the host cell were identified as a class II holin with two transmembrane domains in an N-in, C-in topology and an *N*-acetylmuramoyl-l-alanine amidase, respectively.

Several genes of interest were found, including those encoding an FtsK/SpoIIIE protein, a phage Mu Mom-like protein, and a DnaJ heat shock protein. In spore-forming bacteria, SpoIIIE is an ATP-dependent DNA transporter used to pump DNA into the forespore during sporulation (9). The Mom protein, found in phage Mu, is a sequence-specific adenosine methylase that protects phage DNA from restriction endonucleases (10). Mom regulatory proteins C and Com have yet to be identified in Riggi. A protein containing a cysteine-rich zinc finger domain, commonly found in DnaJ-like chaperone proteins, that contains two conserved C-terminal CXXCXGXG motifs (11) was also identified.

### Nucleotide sequence accession number.

The genome sequence of phage Riggi was contributed to GenBank with the accession number KF669659.
